# RNA Chaperone Function of a Universal Stress Protein in *Arabidopsis* Confers Enhanced Cold Stress Tolerance in Plants

**DOI:** 10.3390/ijms18122546

**Published:** 2017-11-27

**Authors:** Sarah Mae Boyles Melencion, Yong Hun Chi, Thuy Thi Pham, Seol Ki Paeng, Seong Dong Wi, Changyu Lee, Seoung Woo Ryu, Sung Sun Koo, Sang Yeol Lee

**Affiliations:** Division of Applied Life Science (BK21+ Program), PMBBRC, Gyeongsang National University, Jinju 52828, Korea; sarah_melencion@yahoo.com (S.M.B.M.); gandhi37@gnu.ac.kr (Y.H.C.); phamthuy1107ls@gmail.com (T.T.P.); skpaeng@gmail.com (S.K.P.); wsd3377@gmail.com (S.D.W.); royce7457@gnu.ac.kr (C.L.); asd7030@naver.com (S.W.R.); moomoo3837@gmail.com (S.S.K.)

**Keywords:** *Arabidopsis thaliana* universal stress protein (AtUSP), DNA- and RNA-binding activity, nucleic acid-melting activity, anti-termination activity, enhanced cold tolerance, RNA chaperone

## Abstract

The physiological function of *Arabidopsis thaliana* universal stress protein (AtUSP) in plant has remained unclear. Thus, we report here the functional role of the *Arabidopsis* universal stress protein, AtUSP (At3g53990). To determine how AtUSP affects physiological responses towards cold stress, AtUSP overexpression (AtUSP OE) and T-DNA insertion knock-out (*atusp*, SALK_146059) mutant lines were used. The results indicated that AtUSP OE enhanced plant tolerance to cold stress, whereas *atusp* did not. AtUSP is localized in the nucleus and cytoplasm, and cold stress significantly affects RNA metabolism such as by misfolding and secondary structure changes of RNA. Therefore, we investigated the relationship of AtUSP with RNA metabolism. We found that AtUSP can bind nucleic acids, including single- and double-stranded DNA and luciferase mRNA. AtUSP also displayed strong nucleic acid-melting activity. We expressed AtUSP in RL211 *Escherichia coli*, which contains a hairpin-loop RNA structure upstream of chloramphenicol acetyltransferase (*CAT*), and observed that AtUSP exhibited anti-termination activity that enabled *CAT* gene expression. AtUSP expression in the cold-sensitive *Escherichia coli (E. coli)* mutant BX04 complemented the cold sensitivity of the mutant cells. As these properties are typical characteristics of RNA chaperones, we conclude that AtUSP functions as a RNA chaperone under cold-shock conditions. Thus, the enhanced tolerance of AtUSP OE lines to cold stress is mediated by the RNA chaperone function of AtUSP.

## 1. Introduction

Plants are continually exposed to detrimental environmental factors such as extreme temperature, water deficit, salinity, heavy metals, pathogens, and herbivore attack. Environmental stresses can delay plant development and reduce growth. Plants have evolved strategies to detect and adapt to environmental stresses to improve survival and reproduction and to regulate molecular, cellular, and physiological responses to stress [1,2,3]. Many stress-responsive genes have been identified that have essential roles in defense responses against environmental stresses.

The first universal stress protein (USP) identified was C13.5 protein in bacteria; its gene expression was stimulated by a wide variety of stresses. USPs have essential roles for bacterial survival under abiotic stresses such as nutrient starvation, high salinity, extreme temperatures, and chemical exposure [4]. In *Escherichia coli*, USP has six paralogs designated as UspA, C, D, E, F, and G. Bacterial USPs are involved in processes such as cell motility, cell adhesion, iron scavenging, and oxidative stress resistance [5]. USPs have been identified in fungi, archaebacteria, eubacteria, protozoa, metazoans, and plants [6].

USP (Pfam accession number PF00582) encodes proteins of 14–15 kDa containing 140–160 highly conserved amino acid residues. USPs have been categorized into two representative groups based on protein structure. One group typically contains five β-strands alternating with four α-helices, and a conserved ATP-binding motif, G-2X-G-9X-G-(S/T). This group is prominent in the USP family of *Methanocaldococcus jannaschii* MJ0557. The other group contains the conserved USP domain but lacks the ATP-binding site. This group is prominent in the USP family of *Haemophilus influenza*. USPs usually contain only the single USP domain or a combination of one or two tandem USP domains along with other functional domains. The additional functional domains can include protein kinase domains, amino acid permease domains, and voltage channel domains. The combination of other domains with the USP domain was proposed to contribute functional diversity to USP responses to a variety of stresses [7,8,9].

*Arabidopsis* contains 44 genes encoding proteins that share high sequence similarity with *E. coli* USPA, which probably evolved from the MJ0577 USP [10,11]. USPs identified in other plant species have essential roles in stress tolerance. The *AtUSP* (At3g53390) gene is significantly induced by salt, osmotic stress, and wounding [12]. Expression of the AtUSP isotypes AtPHOS32 and AtPHOS34 is regulated by microbial elicitors, and the HRU1 isotype is regulated by anoxia [13,14]. Two other *AtUSP* isoforms (At3g62550 and At3g53990) were up-regulated during drought stress [14]. A recent study reports that USP regulates ethylene-mediated signaling and thereby modulates fruit ripening [10]. In addition, other plant species like *Oryza sativa*, OsUSP1 responds to hypoxic conditions [15]. The *USP* genes of *Gossypium arboretum*, *Astragalus sinicus*, *Solanum pennellii*, and *Salicornia brachiate* are involved in water stress and tolerance to drought, salt, and osmotic stress [16,17,18]. We recently reported that AtUSP (At3g53990) OE and *atusp* (SALK_146059) mutant lines displayed a strong tolerant and sensitive phenotypes respectively under oxidative and heat shock stresses compared to control plant suggesting AtUSP functions as a molecular chaperone to protect crucial intracellular macromolecules against oxidative and heat shock stresses [19]. To function as a chaperone, AtUSP protein underwent reversible changes in its tertiary structure in response to external stresses, similar to several other protein chaperones that has been the first discovered as novel and unique property of USP [20,21,22].

*AtUSP* mRNA is strongly induced by cold acclimation in both wild type (WT) *Arabidopsis* [23] and *35S::DREB2A* transgenic plants [24]. However, the physiological and biochemical functions of AtUSP under cold stress have not been elucidated. Here, we investigate the molecular function of AtUSP during cold stress using knock-out (*atusp*) mutant obtained from the ABRC (Arabidopsis Biological Resource Center) and overexpression (AtUSP OE) lines. We show that AtUSP OE plants exhibit enhanced cold stress tolerance compared to WT and *atusp* plants. We also use recombinant AtUSP protein and verify that it functions as an RNA chaperone that enhances cold stress tolerance in plants.

## 2. Results

### 2.1. Transgenic Arabidopsis Overexpressing AtUSP Displays Enhanced Resistance to Chilling and Freezing Stress

**Figure 1:**
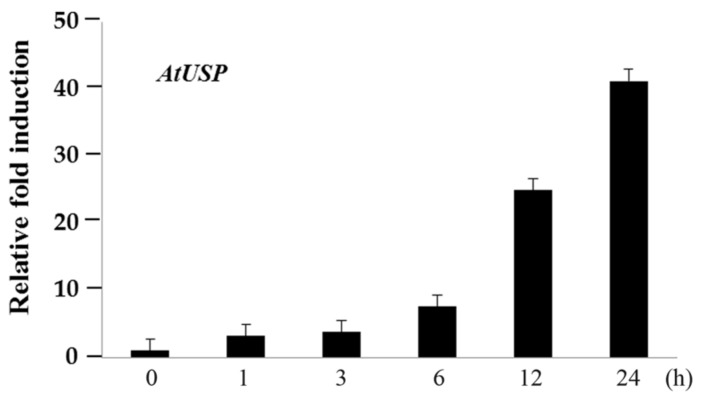
qPCR analyses of *AtUSP* mRNA expression patterns under cold stress conditions in wild type (WT). Ten-day-old WT seedlings grown in MS agar plates were subjected to 4 °C. RNA samples were collected at 0, 1, 3, 6, 12, and 24 h, qPCR analyses were performed using specific AtUSP primers. Relative *AtUSP* mRNA expression levels were determined with respect to the relative abundance of *Actin* and *Ubiquitin1* mRNA. All values are means for three replicates ± SE.

We used the Bio-Analytic Resource (BAR) for plant biology bioinformatics database (Available online: http://bar.utoronto.ca/) to analyze *AtUSP* mRNA transcript levels in *Arabidopsis*, and found that they are significantly up-regulated by cold treatment (4 °C) (Figure S1 of Supplemental Materials). We confirmed the expression level of *AtUSP* expression levels under the cold stress by performing real-time PCR (RT-PCR) and quantitative PCR (qPCR) analyses. The transcript levels in 10-day-old *Arabidopsis* seedlings increased up to 40-fold within 24 h after cold treatment (4 °C) compared to control plants (Figure 1 and Figure S2 of Supplemental Materials). These results suggest that AtUSP has a crucial role in cold-shock resistance in plants.

**Figure 2:**
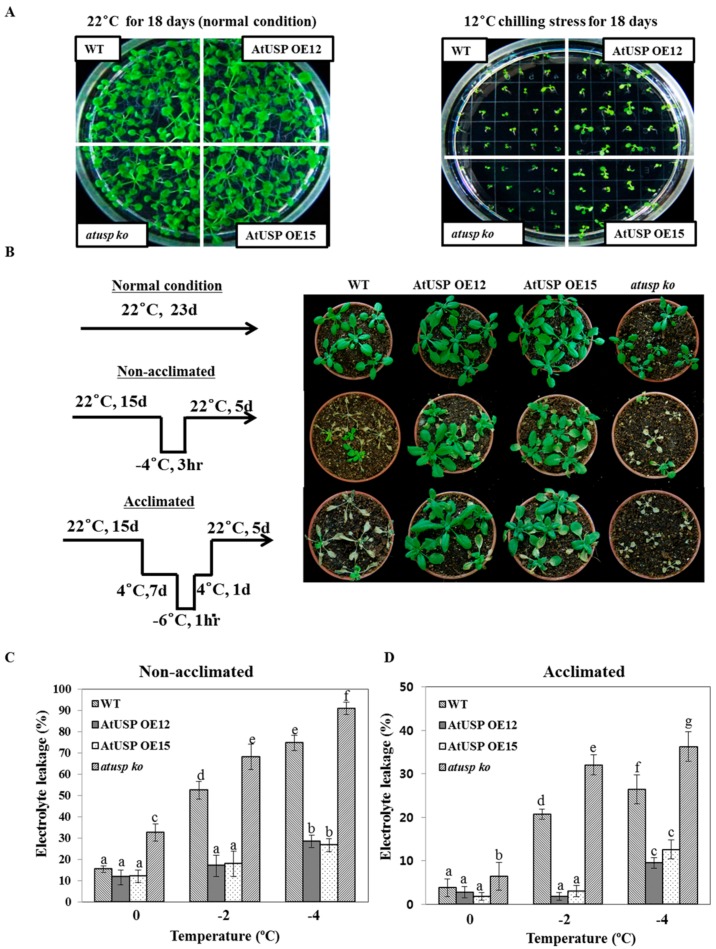
Chilling and freezing tolerance of *atusp* knock-out and transgenic plants overexpressing AtUSP. (**A**) Effect of chilling stress at 12 °C in WT, AtUSP OE, and *atusp* plants. Three-day-old seedlings grown on 1/2 MS agar plates were subjected to 12 °C chilling stress for 18 days, and then phenotypes were assessed. ((**B**), **left panel**) The scheme of cold treatment of WT, AtUSP OE, and *atusp* plants were treated with cold temperatures as shown in the schematic (**left panel**). For non-acclimated cold treatment, 15-day-old seedlings grown in soil at 22 °C were transferred to −4 °C for 3 h, and then returned to 22 °C for recovery. For acclimated cold treatment, 15-day-old seedlings grown in soil at 22 °C were subjected to 4 °C for 7 days, transferred to −6 °C for 1 h, transferred to 4 °C for 1 day, and finally restored to 22 °C for recovery (**right panel**). The cold-treated *Arabidopsis* plants were photographed after 5 days of recovery. (**C**,**D**) Electrolyte leakage from leaves of WT, AtUSP OE, and *atusp* plants after exposure to temperature ranges from 0 to −4 °C. Simplified electrolyte leakage assay was described in Figure S3 of Supplemental Materials and Materials and Methods. Experiments were performed with at least three biological replicates. All values are means for three replicates ± SE. To compare differences between treatments, data were analyzed using one-way ANOVA and Tukey’s test. Different letters indicate significant differences among the plant lines (*p* < 0.05).

We investigated the physiological and functional roles of AtUSP under cold stress conditions using transgenic overexpressing (AtUSP OE, lines 12 and 15) and knock-out (*atusp*) lines prepared in our laboratory along with WT control [19]. The result showed that all lines incubated at 22 °C grew well with no phenotypical differences among WT, AtUSP OE, and *atusp* plants (Figure 2A, left panel). By contrast, plants incubated at 12 °C displayed significant growth differences (Figure 2A, right panel). AtUSP OE plants enhanced the resistance of plants to cold treatment, whereas *atusp* mutant plants displayed growth defects and sensitivity within 18 days of cold treatment. To further confirm the cold tolerance and sensitivity of overexpression and knock-out lines, we analyzed their growth phenotypes in soil with and without acclimation to freezing conditions (Figure 2B, left panel). As a result, AtUSP OE plants recovered growth after freezing stress under both non-acclimated and acclimated conditions (Figure 2B, right panel). By contrast, most of the WT and *atusp* plants did not recover from freezing stress even with cold acclimation, indicating that these plants are cold-sensitive. We analyzed membrane damage in acclimated and non-acclimated plants subjected to cold-shock treatment by measuring electrolyte leakage. Consistent with their phenotypic data (Figure 2B, right panel), AtUSP OE plants had significantly lower electrolyte leakage than WT or *atusp* plants (Figure 2C,D). These combined results suggest that the *Arabidopsis* USP has a crucial functional role in protection from cold/freezing shock, and USP overexpression enhances cold-shock resistance in plants.

### 2.2. Subcellular Localization of AtUSP

**Figure 3:**
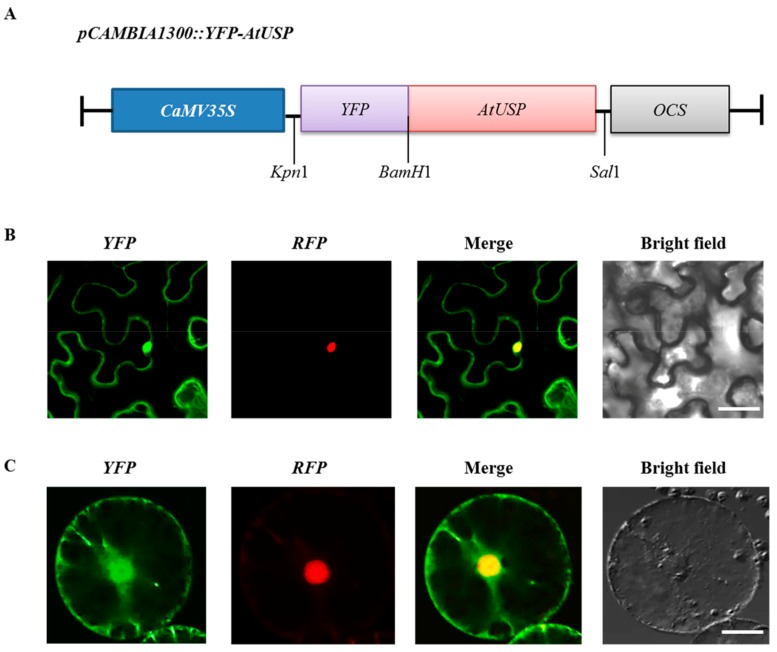
Subcellular localization of AtUSP in tobacco leaves and protoplasts. (**A**) Schematic representation of the YFP-AtUSP construct. *CAMV35s* and *OCS* indicate Cauliflower mosaic virus 35S promoter and octopine synthase terminator, respectively. (**B**) Confocal images of tobacco leaf abaxial epidermis and (**C**) isolated protoplast following co-transfection with the fusion protein constructs. From left to right: YFP and red fluorescent protein (RFP) (nuclear localization signal (NLS)-RFP) fluorescence, merged image of YFP and RFP (merge), and bright field image. NLS-RFP (chimeric protein with the simian virus 40 (SV40) large T antigen NLS fused N-terminal RFP) was used as nuclear marker protein. Scale bar = 20 μm.

To elucidate the functional role of AtUSP during cold adaptation, we analyzed the subcellular location of AtUSP in tobacco. Before doing the experiment, we confirmed that AtUSP did not contain the chloroplast transit peptide or mitochondrial targeting presequence by using ChloroP 1.1 server (Available online: http://www.cbs.dtu.dk/services/ChloroP/) and MITOPROT (Available online: https://ihg.gsf.de/ihg/mitoprot.html). Based on the result, it was possible to construct the *pCAMBIA1300::YFP-AtUSP* fusion construct (Figure 3A) and transiently expressed it in tobacco leaves (Figure 3B). YFP-AtUSP (AtUSP tagged with yellow fluorescent protein) fluorescence was observed in the tobacco nucleus and cytoplasm. The nuclear fluorescence signal clearly overlapped with the nuclear localization signal (NLS) tagged with red fluorescent protein (NLS-RFP). This result confirmed that AtUSP was localized in the nucleus (Figure 3B). To further examine the nuclear localization of AtUSP, we isolated tobacco protoplasts and confirmed the co-localization of YFP-AtUSP and NLS-RFP (Figure 3C). These results suggest that AtUSP is localized to the plant nucleus and cytoplasm and may play an important role in nucleus and cytoplasm compartments under cold stress condition.

### 2.3. DNA- and RNA-Binding Activity of AtUSP

Next, we evaluated the molecular mechanism underlying USP-mediated tolerance to cold shock. RNAs are predominantly distributed in the nucleus and cytoplasm, and cold shock induces RNA stabilization into misfolded and non-specific hairpin structures [25]. Furthermore, the USP domain is reported to be involved in nucleotide binding, DNA protection, and DNA repair [4,26,27,28]. Therefore, we analyzed the interactions of AtUSP with RNA and DNA.

Cold-shock resistance is typically provided by RNA chaperones, which have several shared characteristics: binding activity for single-stranded DNA (ssDNA), double-stranded DNA (dsDNA), and *luciferase* mRNA; nucleic acid-melting activity; anti-termination activity of RNA transcription; and complementation of the cold-sensitive *E. coli* mutant BX04 [29,30,31,32,33]. Therefore, we investigated whether AtUSP exhibits similar characteristics as RNA chaperones by analyzing the DNA- and RNA-binding activity of recombinant AtUSP. We used M13 mp8 phage to isolate ssDNA and dsDNA, and *luc* mRNA.

**Figure 4:**
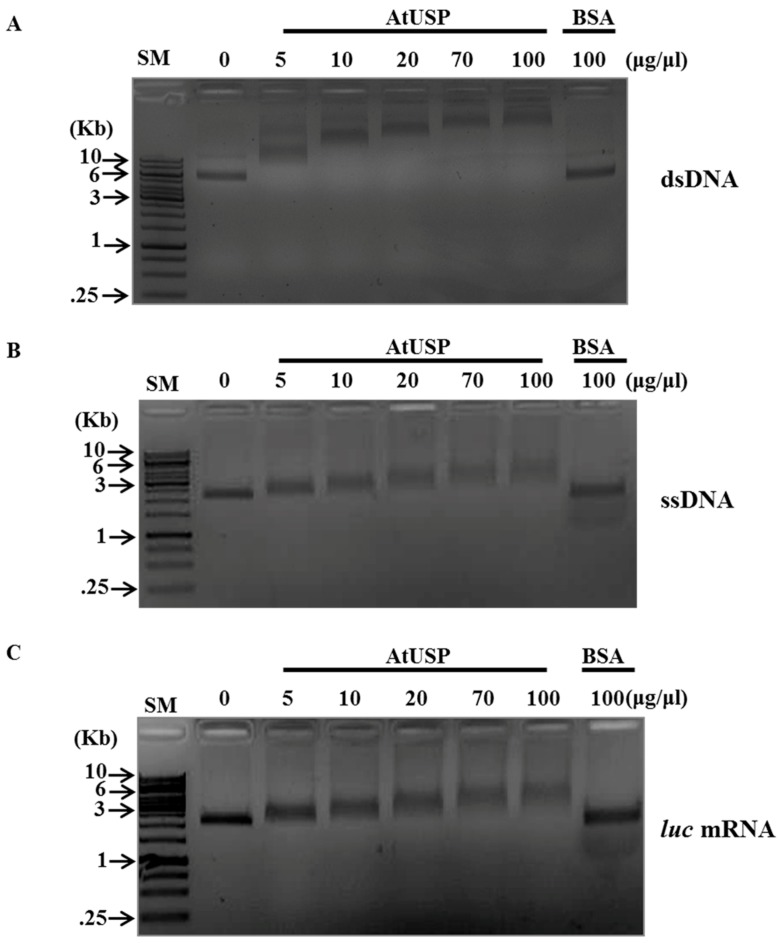
Nucleic acid-binding activity of AtUSP in vitro. Indicated amounts of purified recombinant AtUSP protein were incubated with either (**A**) M13mp8 ssDNA, (**B**) M13mp8 dsDNA, or (**C**) in vitro transcribed luciferase (*luc*) mRNA. To analyze the effect of AtUSP in RNA mobility and the AtUSP-RNA complexes, 0.8% agarose gels were used for gel-shift assays. Bovine serum albumin (BSA) protein (100 μg/μL) was used as a negative control. SM presents size marker from Thermo Scientific Company.

We incubated various concentrations of purified AtUSP recombinant protein with the respective substrates in reactions containing binding buffer, and then performed gel-shift assays using 0.8% agarose gels (Figure 4). Increasing AtUSP concentrations correspondingly showed successive retardation of nucleotide migration with dsDNA, ssDNA, and *luc* mRNA in the agarose gels, presumably due to higher levels of bound AtUSP (Figure 4A–C). By contrast, excessive concentrations (100 μg/μL) of bovine serum albumin (BSA) negative control did not change the substrate migration, indicating that AtUSP specifically binds to ssDNA, dsDNA, and RNA. These combined results suggest that AtUSP displays DNA- and RNA-binding activity similar to that of RNA chaperones.

### 2.4. Nucleic Acid-Melting Activity of AtUSP

**Figure 5:**
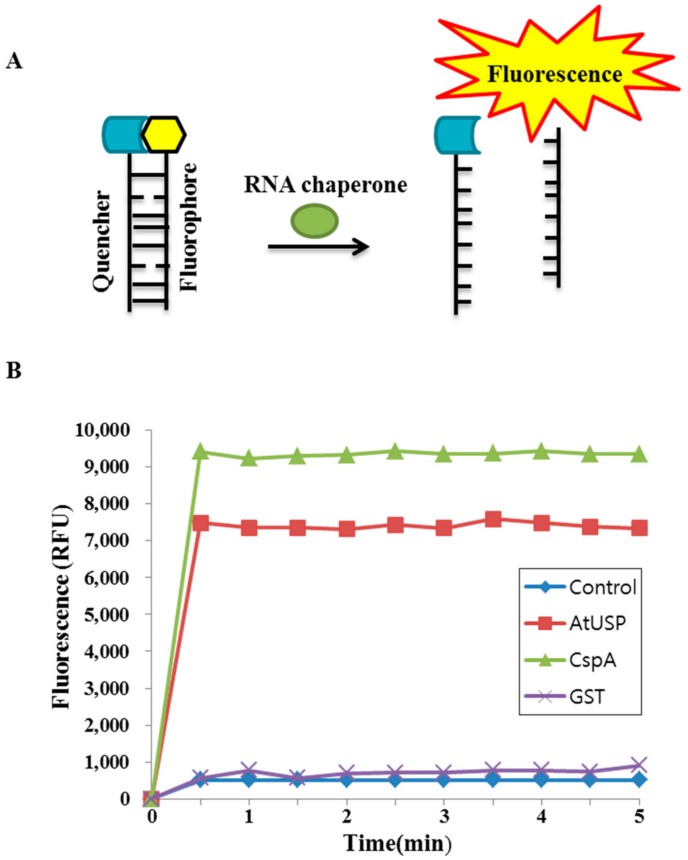
Nucleic acid-melting activity of AtUSP using a molecular beacon substrate. (**A**) Schematic diagram of DNA-melting activity of AtUSP, which was analyzed using a molecular beacon substrate. F, fluorophore (FITC); Q, fluorescence quencher. (**B**) Effect of AtUSP, cold-shock protein A (CspA), and GST on DNA melting using a molecular beacon substrate. Emitted fluorescence intensity was measured at various times after incubating AtUSP, CspA, GST, and buffer alone (control) with the molecular beacon. Excitation and emission wavelengths were 555 and 575 nm, respectively.

We examined whether AtUSP displays nucleic acid-melting activity using two partially complementing oligonucleotides as a molecular beacon substrate. One strand of the substrate was labeled with FITC at the 5′ terminus, and the other strand was labeled with the fluorescent quencher BHQ1 at the 3′ terminus. The molecular beacon containing fluorophore and quencher formed a stem-loop structure at the end, and FITC fluorescence is efficiently quenched when the two ends of the molecular beacon remain annealed (Figure 5A). However, when RNA chaperone binds to and melts this stem-loop structure, the quencher becomes spatially separated and fluorescence is emitted (Figure 5A).

We tested the nucleic acid-melting activity of AtUSP by incubating the AtUSP recombinant protein with the molecular beacon and measuring the emitted fluorescence intensity. Cold-shock protein A (CspA) and glutathione *S*-transferase (GST) were used as positive and negative controls, respectively. The results showed that AtUSP significantly increased the fluorescence intensity of the molecular beacon to a level slightly lower than that of the CspA positive control (Figure 5B). By contrast, GST did not increase the molecular beacon’s fluorescence intensity (Figure 5B). These combined results indicate that AtUSP displays nucleic acid-melting activity in vitro.

### 2.5. AtUSP Exhibits Anti-Termination Activity in the Transcription of Bacterial Chloramphenicol Acetyltransferase (CAT)

**Figure 6:**
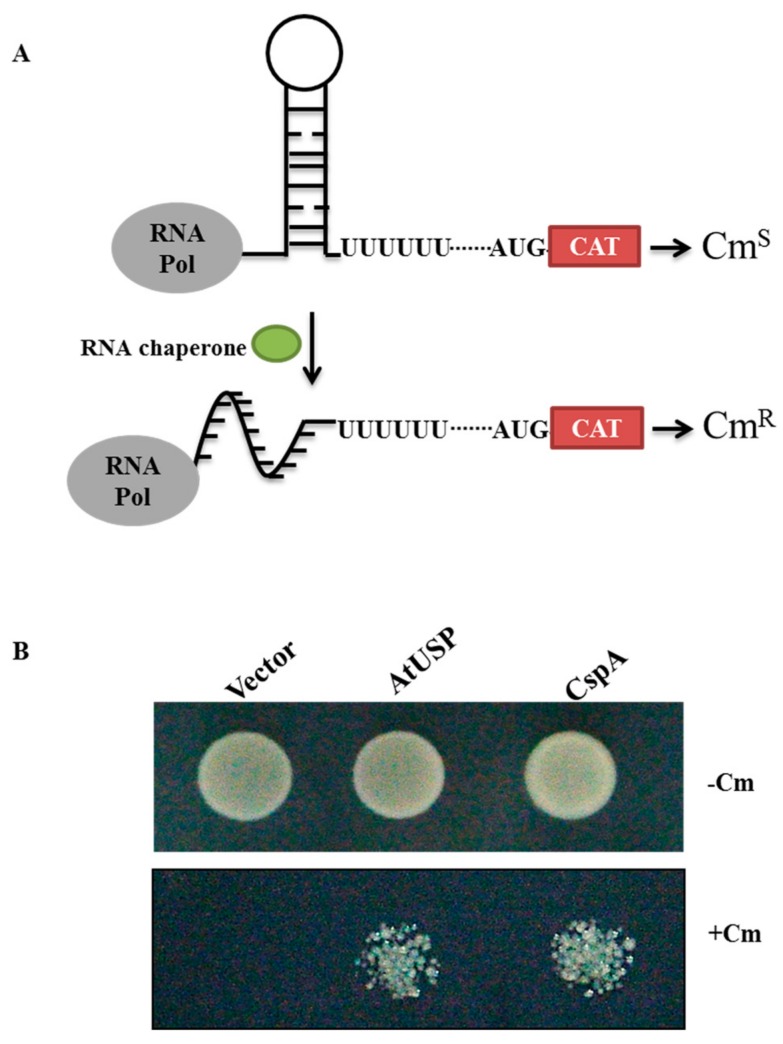
AtUSP displays anti-termination activity in chloramphenicol acetyltransferase *(CAT)* gene transcription in RL211 *E. coli*. (**A**) Schematic representation for the measurement of AtUSP anti-termination activity using RL211 *E. coli* cells, which contain a stem-loop RNA structure upstream of the chloramphenicol (*CAT*) gene. AtUSP can melt the stem-loop RNA hairpin structure, which results in *CAT* gene transcription and chloramphenicol (Cm) resistance. (**B**) *pINIII* vector, *pINIII-AtUSP*, or *pINIII-CspA* were transformed into RL211 cells harboring a chloramphenicol resistance gene downstream of the trpL terminator. Cells were incubated in media containing 0.5 mM isopropyl-β-d-thiogalacto-pyranoside (IPTG) with or without Cm. Equal volumes of cell cultures were spotted onto LB-carbenicillin agar medium with or without Cm and grown at 37 °C.

RNA chaperones melt hairpin-loop RNA structures [34,35]. Therefore, we evaluated the anti-termination activity of recombinant AtUSP using RL211 cells harboring the *CAT* gene upstream of Rho-independent trpL terminator. The sliding of the small ribosomal subunit is blocked due to RNA secondary structure upstream of the AUG. The transcription Rho-terminator stem fold cause transcription termination and inhibits *CAT* gene expression allowing the cells to be sensitive under chloramphenicol treatment (Figure 6A). We cloned *AtUSP* into the inducible *pINIII* expression vector and construct was transformed into RL211 cells. RL211 cells expressing AtUSP were grown on LB-agar medium containing chloramphenicol. The *pINIII* and *CspA* gene constructs were selected as negative and positive controls, respectively. RL211 cells expressing *pINIII* did not grow on chloramphenicol plates, whereas those expressing AtUSP and CspA grew well under chloramphenicol selection (Figure 6B). This result indicates that AtUSP melted the secondary stem-loop structure in the RNA termination region in vivo, which enabled *CAT* gene expression and cell growth in medium supplemented with chloramphenicol, which is consistent with the function of an RNA chaperone.

### 2.6. AtUSP Complements Cold-Shock Sensitive BX04 E. coli Mutant Cells

**Figure 7:**
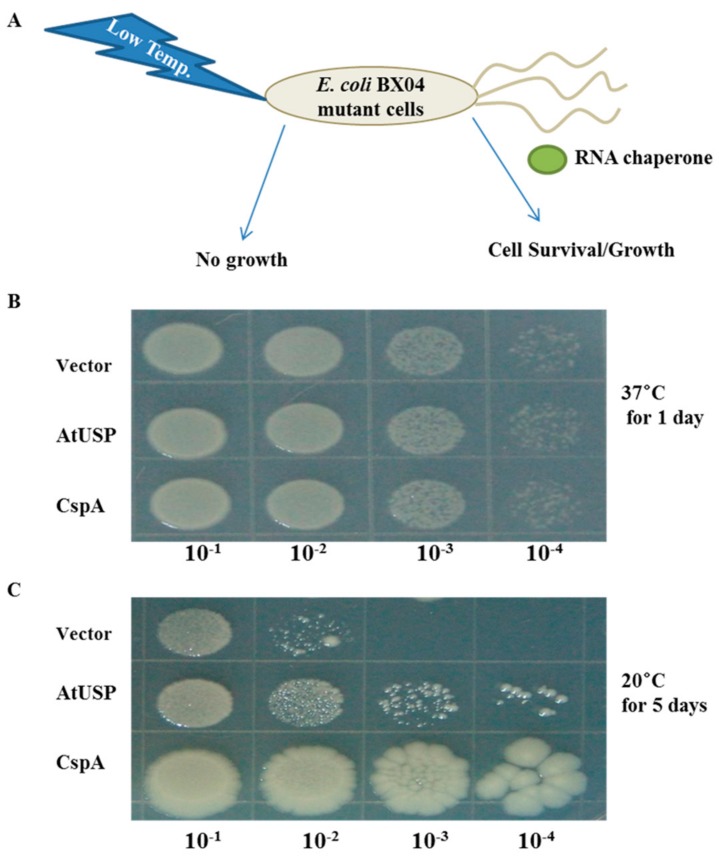
AtUSP complements cold-sensitive *E. coli* BX04 mutants. (**A**) Schematic diagram of the cold-shock assay in *E. coli*. BX04 mutants (Csp-deficient *E. coli*) lack RNA chaperone genes and are highly sensitive to low temperature. However, cells expressing an RNA chaperone can grow under the same cold-shock conditions. (**B**,**C**) Diluted aliquots (10^−1^ to 10^−5^) of BX04 culture cells expressing *pINIII*, *pINIII-AtUSP*, or *pINIII-CspA* were uniformly spotted onto LB-carbenicillin plates and grown at either 37 or 20 °C. Pictures were taken after incubating for 1 day at 37 °C (**B**) and 5 days at 20 °C (**C**).

To elucidate the molecular mechanism of AtUSP-mediated cold-shock tolerance (Figure 2), we evaluated whether AtUSP complements the cold-sensitive phenotype of BX04 *E. coli* mutants, which lack the four essential cold-resistant *Csp* genes (*CspA/B/E/G*) [36]. *E. coli* BX04 mutants cannot survive cold-shock treatment, but do grow and survive the same conditions when complemented by RNA chaperone protein expression (Figure 7A). Therefore, we transformed *pINIII*, *pINIII-AtUSP*, and *pINIII-CspA* constructs into BX04 quadruple mutant cells, incubated the transformants at different temperatures, and evaluated colony survival on LB plates. All BX04 transformants grew well at 37 °C and did not display significant growth differences (Figure 7B). At 20 °C (low temperature), transformants expressing *pINIII* vector alone failed to grow well, whereas those expressing either AtUSP or CspA grew much better (Figure 7C). These results suggest that AtUSP can complement BX04 mutants and protect against cold-shock conditions.

The combined results of Figure 2, Figure 3, Figure 4, Figure 5, Figure 6 and Figure 7 indicate that AtUSP functions as an RNA chaperone under cold stress conditions in plant and bacterial cells, which enables organisms to tolerate and survive low temperatures. The cold-tolerant phenotype of *Arabidopsis* overexpressing AtUSP (Figure 2) is likely conferred by the RNA chaperone function of AtUSP under cold-shock conditions.

## 3. Discussion

RNAs are crucial for cellular regulatory processes. However, kinetic and thermodynamic problems can arise during RNA folding and formation of the tertiary structure. Environmental stresses cause the formation of non-functional and misfolded RNAs. For example, cold stress conditions induce highly stable and inactive RNA structures. RNA chaperones disrupt misfolded RNAs and assist with refolding so that they attain their native, active states. These refolded RNAs perform normal functions including RNA metabolism, translation, mRNA splicing, and RNA decay, even under stress conditions. The functional roles of RNA chaperones have been well defined when cells are subjected to low temperature [36,37,38,39,40,41].

**Figure 8:**
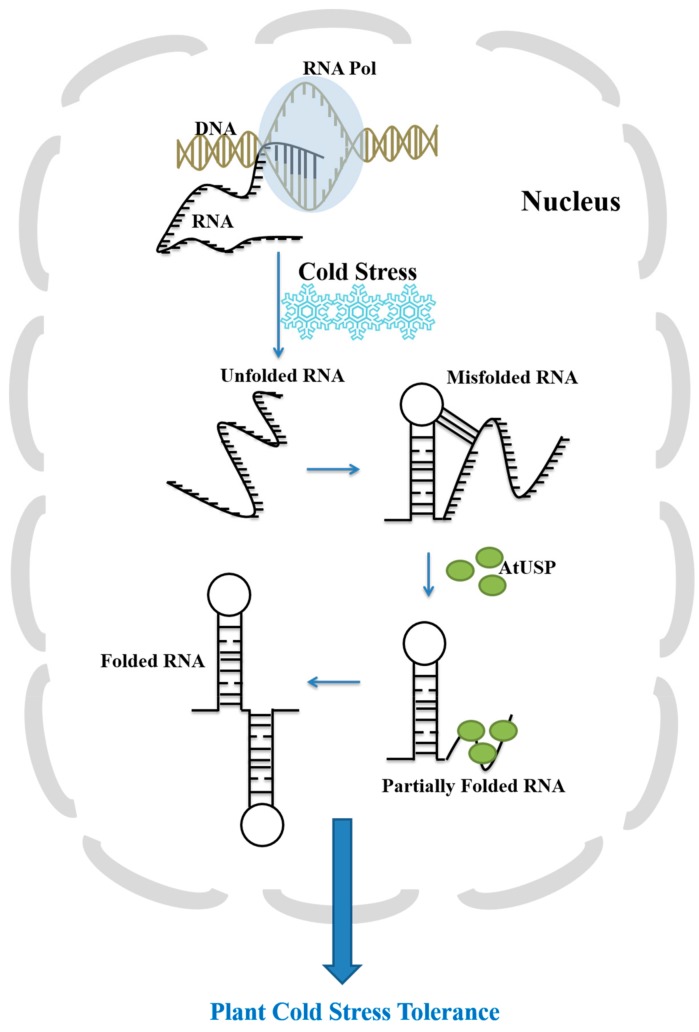
Proposed model of the RNA chaperone function of AtUSP. When plants are exposed to cold stress, the molecular structure of RNAs can be over-stabilized to form non-native, misfolded, and inactive conformations. AtUSP acts as an RNA chaperone and binds to the misfolded RNAs, melts the inactive conformations, and assists refolding into the native, active RNA conformations. Then, the active RNAs can be expressed to produce cold shock-resistant proteins, which enhances cold tolerance in plants. RNA Pol indicates RNA polymerase.

We previously reported that AtUSP has an important role as a protein molecular chaperone under heat shock or oxidative stress conditions [19]. The BAR bioinformatics database indicates that *AtUSP* is strongly induced during cold acclimation [23,24]. However, the physiological and molecular functions of AtUSP remained to be elucidated. In the present study, we discovered a novel function of AtUSP: to act as an RNA chaperone under cold-shock conditions. Normally, RNA molecules are incorporated as single-stranded poly-anionic chains but when cold and freezing temperatures induced, RNA misfolds into non-functional conformations. AtUSP functions as an RNA chaperone to destabilize RNA secondary structure and mediate efficient refolding into active conformations (Figure 8). AtUSP binds nucleic acids (ssDNA, dsDNA, and RNA). Under cold conditions, AtUSP also promotes RNA transcription and translation, mediates nucleic acid-melting, and displays anti-terminating gene transcription activity. Similar to the nucleic acid-melting of rice OsZR2, OsGRP4 and OsGRP6, AtUSP can melt RNA secondary structures, which correlates with its in vivo anti-termination activity [33]. Therefore, the RNA chaperone function of AtUSP confers enhanced cold tolerance against cold and freezing stress in plants. AtUSP also complements the cold-sensitive phenotype of BX04 *E. coli* mutant cells, confirming that AtUSP functions as a RNA chaperone and confers cold and freezing tolerance in *E. coli*, similar to the rice RNA chaperones OsGRP4 and OsGRP6 [30,31,32].

Many RNA chaperones contain common motifs such as the cold-shock domain (CSD), Cys-Cys-His-Cys (CCHC) zinc finger motif, RNA recognition motif (RRM), and glycine-rich region (GRR). Although AtUSP displays RNA chaperone functions, it does not contain the conserved motif or domains. Recent work identified several RNA chaperones that lack common motifs or domains, such as hPrx1 [42], which destabilizes misfolded RNAs. Similar to the other RNA chaperones, such as WCSP1 from wheat and AtGRPs from *Arabidopsis*, AtUSP ligated to pINIII vector, pINIII-AtUSP, enables the *E. coli* RL211 cells to grew on chloramphenicol plates (Figure 6). Furthermore, the nucleic acid-melting activity assayed with the use of recombinant AtUSP protein can also melt the beacon substrate (Figure 5), which can be a direct evidence for its RNA chaperone function. From these results, it can be concluded that AtUSP exhibit RNA chaperone function in organisms.

Most RNAs and RNA chaperones are located in the cytosol and the nucleus to regulate RNA import, export, and processing [23,24]. For the reaction, RNA chaperones should be reversibly translocated between the nucleus and cytoplasm, which requires the NLS and NES sequences in coding region of the protein. Consistent with the subcellular localization of WCSP1a, a RNA chaperone [25], we also observed that AtUSP was distributed both in the nucleus and cytoplasm (Figure 3). Based on the result, we searched the NLS and NES sequences of AtUSP by in silico analysis. From the searches, it was possible to detect the putative NLS motif of AtUSP at the region of ^92^RQKEVHVVTKLYWGDAREKLVDAVKDLKLD^121^ by using the cNLS Mapper database (Available online: http://nls-mapper.iab.keio.ac.jp/cgi-bin/NLS_Mapper_form.cgi). In addition, the NetNES 1.1 Server (Available online: http://www.cbs.dtu.dk/services/NetNES/) provided us the NES sequence of AtUSP at its C-terminal region, ^118^LKLDSI^123^. The dual-targeting of AtUSP both in the nucleus and cytoplasm provides us the possibility that AtUSP can play highly important roles in the regulation of abiotic stress dynamically changing its subcellular compartments between the nucleus and the cytoplasm. Moreover, AtUSP also was detected in the plasma membrane (PM) and ER. The specific functions of AtUSP in the PM and ER should be investigated in future experiments.

Recent studies report that RNA chaperones have significant roles in plant growth and development [27]. For example, AtCSP2 and AtRH3 (chloroplast-localized DEAD-box RNA helicase) are involved in multiple steps of chloroplast development and plant growth, respectively [32,36]. The AtGRP4 and AtRZ-1a was identified to affects the seed germination and seedling growth under low temperature in *Arabidopsis* plants [43,44]. RNA chaperones also function in multi-stress resistance in plants, as shown for GRP and RZ family members including AtGRP4 and AtGRP7 in *Arabidopsis*, OsZR2 in rice, and WCSP1 in wheat [30,31,32]. The AtUSP promoter is co-regulated by phytohormones and multiple abiotic stresses [45]. Therefore, AtUSP play a role in diverse abiotic stresses and phytohormone signaling pathways but further study should be conducted. In addition, there are about 44 USP-homolog genes in the *Arabidopsis* genome [11,12,45]. However, the physiological function of the genes has not almost been identified, which should be done in future.

In conclusion, AtUSP displays similar functions as RNA chaperones, including nucleotide binding, nucleotide melting, anti-termination activity, and complementation of the cold-sensitive BX04 mutant. These combined results indicate that AtUSP functions as an RNA chaperone under cold stress conditions. The discovery of this novel function provides a new biotechnological target to improve crop yields under cold conditions.

## 4. Materials and Methods

### 4.1. RT-PCR and qPCR Analysis of mRNA Expression

Ten-day-old seedlings were subjected to cold-shock treatment at 4 °C and samples were collected at 0, 1, 3, 6, 12, and 24 h. The samples were frozen in liquid nitrogen and used for RNA isolation and cDNA synthesis. Total RNA was extracted from the frozen samples using the MACHEREY-NAGEL RNA kit (Düren, Germany). Isolated RNA was reverse-transcribed using the RevertAid Reverse Transcriptase and First-Strand Synthesis kit (Thermo Scientific, Vilnius, Lithuania) according to the manufacturer’s instructions. The newly synthesized cDNA was diluted to 50 ng/μL with ddH_2_O. The following PCR program was used: 95 °C denaturation for 2 min; 24 cycles of 95 °C for 20 s, 60 °C for 40 s, and 72 °C for 1 min; and then an elongation step at 72 °C for 5 min. Specific PCR primers for *AtUSP* genes were as follows: *AtUSP* forward, 5′-GAATTCCATGCCTAAAGACAGGAATATCGG-3′; AtUSP reverse, 5′-ATCGATTTATTCGTTATCCTTGACAACGGT-3′. *AtUSP* gene expression levels were compared with those of the internal control gene *Tubulin* (AT5G62690). Specific PCR primers for *Tubulin* genes (AT5G62690) were as follows: forward, 5′-CCAACAACGTGAAATCGACA-3′; reverse, 5′-TCTTGGTATTGCTGGTACTC-3′. PCR products were observed in 1% agarose gels stained with ethidium bromide. Real-time quantitative PCR (RT-qPCR) was performed with CFX Touch™ Real-Time PCR Detection System (Biorad, Hercules, CA, USA) using the TOP Real™ qPCR @X Pre MIX (SYBR Green with high ROX) Kit (Enzynomics, Daejeon, Korea) according to the manufacturer’s protocol. PCR cycling conditions were as follows: 95 °C for 15 min; 40 cycles of 95 °C for 10 s, 55 °C for 10 s, and 72 °C for 3 s; followed by a melting curve step for checking the specificity of the amplified products. Three biological replicates for each sample were performed, and expression levels were normalized by using Actin2 and *Ubiquitin1*. Specific PCR primers for RT-qPCR analysis of *AtUSP*, *Actin*, and *Ubiquitin1* were as follows: for AtUSP, forward, 5′-TATCGGAATCGCCATGGATT-3′; and reverse, 5′-TCTCGATCGCCCATTTCAG-3′; for *Actin2*, forward, 5′-TGATGCACTTGTGTGTGACAA-3′; reverse, 5′-GGGACTAAAACGCAAAACGA-3′; for *Ubiquitin1*, forward, 5′-TTCCTTGATGATGCTTGCTC-3′; reverse, 5′-TTGACAGCTCTTGGGTGAAG-3′.

### 4.2. Chilling and Freezing Tolerance Assay

The freezing tolerance assay was performed as described previously [46] with modifications. Briefly, 3-day-old seedlings of WT, AtUSP OE, and *atusp* knock-out mutants grown in MS were transferred to 12 °C for 18 days, and then phenotypes were assessed. For acclimation assays, all seedlings were grown in soil in a growth chamber at 22 °C for 15 days, incubated at 4 °C for 7 days, transferred to −6 °C for 1 h, transferred to 4 °C for 1 day, and then transferred back to 22 °C for 5 days for recovery. For non-acclimation assays, seedlings were grown in soil in a growth chamber and transferred to 22 °C. After 15 days, some plants were transferred directly to −4 °C for 3 h, and then transferred back to 22 °C for recovery.

### 4.3. Electrolyte Leakage Test

The electrolyte leakage test was performed as described previously [31]. Briefly, the 5th and 6th leaves of 15 days old plants *Arabidopsis* were stored in a test tube containing 100 μL distilled water, and the tube was incubated for 1 h in a controlled-temperature circulating water bath at 0 °C. Ice crystals were added to the tube as water bath temperature was programmed to −4 °C at a rate of 1 °C per hour. The test tubes were removed from the water bath when the desired temperature was reached, and conductivity was measured with the conductivity meter. The ratio of electrolyte leakage before and after autoclaving was used as a measure for membrane damage after freezing treatment. The experiment was repeated at least three times.

### 4.4. Subcellular Localization of AtUSP

The full length *AtUSP* and *NLS-RFP* were cloned from an *Arabidopsis* cDNA library and NLS-RFP construct used by Lee et al. [47] respectively by PCR as previously described [19]. After confirmation of sequence, the full length *AtUSP* and *NLS-RFP* were ligated into *BamH*I/*Sal*I sites of the binary vector pCAMBIA1300 with and without YFP in the N-terminal region respectively. The *pCAMBIA1300*::*YFP-AtUSP* and *pCAMBIA1300*::*NLS-RFP* (control) constructs were introduced into *Agrobacterium tumefaciens* strain GV3103, and then used to infiltrate tobacco leaves to express the YFP-AtUSP and NLS-RFP fusion proteins. Infiltrated leaves were incubated in a growth chamber for 2–3 days before observing under a fluorescence microscope (Olympus AX70, Shibuya-ku, Tokyo, Japan) with XF116-2 (exciter, 475AF20; dichroic, 500DRLP; emitter, 510AF23) and U-MWU2 (excitation filter, 330–385; emission filter, 420 nm) filter sets.

### 4.5. Purification of AtUSP Recombinant Protein

The full length cDNA of *AtUSP* was isolated from *Arabidopsis* cDNA library and were ligated into pET28a expreesion vector (NEB) with *BamH*I/*Xho*I sites for the fusion protein construct (pET28a:AtUSP). DNA construct were transform into *E. coli* BL21 (DE3) cells. Transformants were cultured at 37 °C in LB medium with kanamycin 50 μg/mL) and chloramphenicol (12.5 μg/m). The culture were diluted 1:50 in LB medium containing 50 μg/mL kanamycin and grown until the OD reached at OD_600_ of 0.6–0.8 was reached in 30 °C. Recombinant protein expression was induced by the addition of 0.5 mM IPTG and were further grown for 4 h. Then the cells were collected by centrifugation at 5000× *g* for 10 min, an dthe pellet was resuspended with PBS buffer (140 mM NaCL, 2.7 mMKCl, 10mM Na_2_HPO_4_, and 1.8 mM KH_2_PO_4_, pH 7.6) containing 1 mM PMSF. Harvested cells were stored at at −80 °C until use. The frozen cells were sonicated and the soluble extracts was loaded into Ni-NTA agarose columns. The recomninant AtUSP was further eluted from the column by thrombin, and dialyzed against 50 mM Hepes-KOH (pH 8.0) at 4 °C. The recombinant protein was then purified by FPLC using a Superdex 200 HR 10/30 column. The purity of recombinant AtUSP protein with T7 tag was checked by using SDS-PAGE.

### 4.6. Nucleic Acid-Binding Analysis

Gel-shift assays with dsDNA, ssDNA, and *luc* mRNA substrates were performed as described previously [27]. Recombinant AtUSP or BSA was incubated with ssDNA (M13mp18, NEB), dsDNA (M13mp18 RF I DNA, NEB), and *luc* mRNA (TriLink Biotechnologies Co., San Diego, CA, USA) in 15 μL binding buffer (10 mM Tris-HCl, pH 7.5) on ice for 30 min. The samples were then separated using 0.8% agarose gels, and stained with ethidium bromide to visualize migration shifts.

### 4.7. DNA-Melting Assay

Two partially complementing oligonucleotides were labeled with FITC and BHQ1 (black hole quencher). The molecular beacon substrates (FITC:BHQ1 = 1:20) were mixed, denatured at 95 °C for 2 min, and incubated on ice for 20 min. Annealed DNA was used as a molecular beacon substrate for the assay.

### 4.8. Transcription Anti-Termination and Cold Complementation Assay in E. coli

*E. coli* RL211 cells containing a *CAT* gene cassette positioned downstream of the *trpl* terminator were transformed with *pINIII*, *pINIII-CspA*, or *pINIII-AtUSP* and cell were spotted on LB-carbenicillin plates with or without chloramphenicol. Cell growth was inspected at daily intervals. For the cold complementation assay, BX04 mutant cells were transformed with *pINIII*, *pINIII-CspA*, or *pINIII-AtUSP* and grown in LB medium. When the optical density at 600 nm reached approximately 1.0, the cells were spotted on LB-agar plates containing 0.5 mM isopropyl-d-thiogalactopyranoside and were grown under low temperatures [46].

### 4.9. Statistical Analysis

To analyze the data and differences among treatments (*p* < 0.05), we performed one-way ANOVA with Tukey’s test. Statistical analysis was performed using SPSS v. 12.0.1 software (SPSS Inc., Chicago, IL, USA).

## Supplementary Materials

Supplementary materials can be found at www.mdpi.com/1422-0067/18/12/2546/s1.
