# Supplementary material for: RNA Chaperone Function of a Universal Stress Protein in Arabidopsis Confers Enhanced Cold Stress Tolerance in Plants
Source: Int J Mol Sci. 2017 Nov 27;18(12):2546. doi: 10.3390/ijms18122546 (PMC5751149; doi:10.3390/ijms18122546)
Supplement: Supplementary file 1 [file ijms-18-02546-s001.pdf]

Supplementary Materials: RNA Chaperone Function of a Universal Stress Protein in Arabidopsis Confers Enhanced Cold Stress Tolerance in Plants

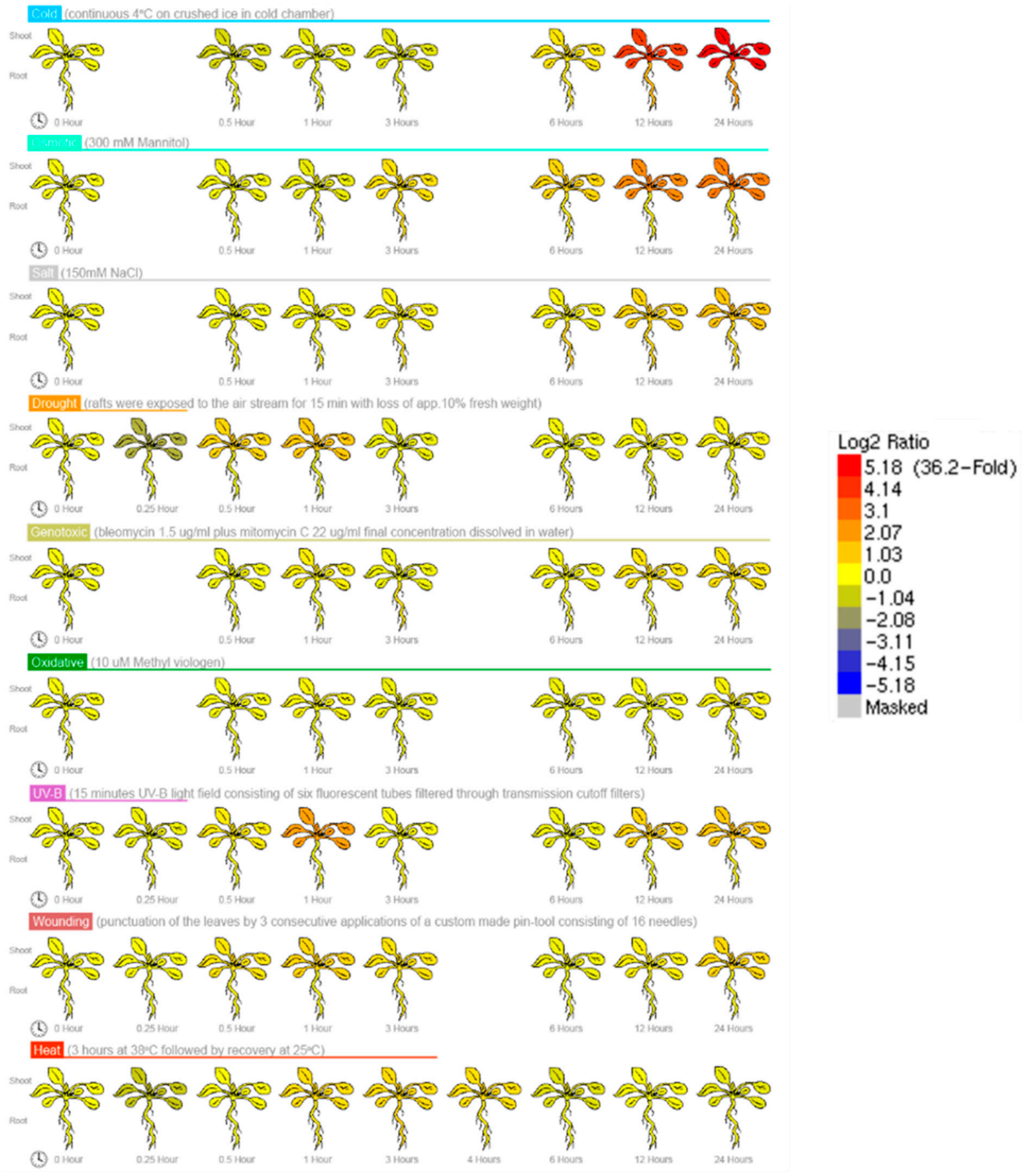

Figure S1. Gene expression pattern of *AtUSP* (At3g53990) under diverse stress conditions analyzed using the eFP browser.

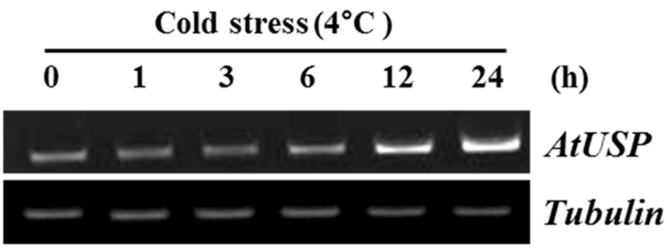

Figure S2. RT-PCR analyses of *AtUSP* mRNA expression patterns under cold stress conditions in WT. Ten-day-old WT seedlings grown in MS agar plates were subjected to 4°C. RNA samples were collected at 0, 1, 3, 6, 12, and 24 h, RT-PCR analyses were performed using specific *AtUSP* primers. Relative *AtUSP* mRNA expression levels were determined with respect to the relative abundance of *Tubulin*.

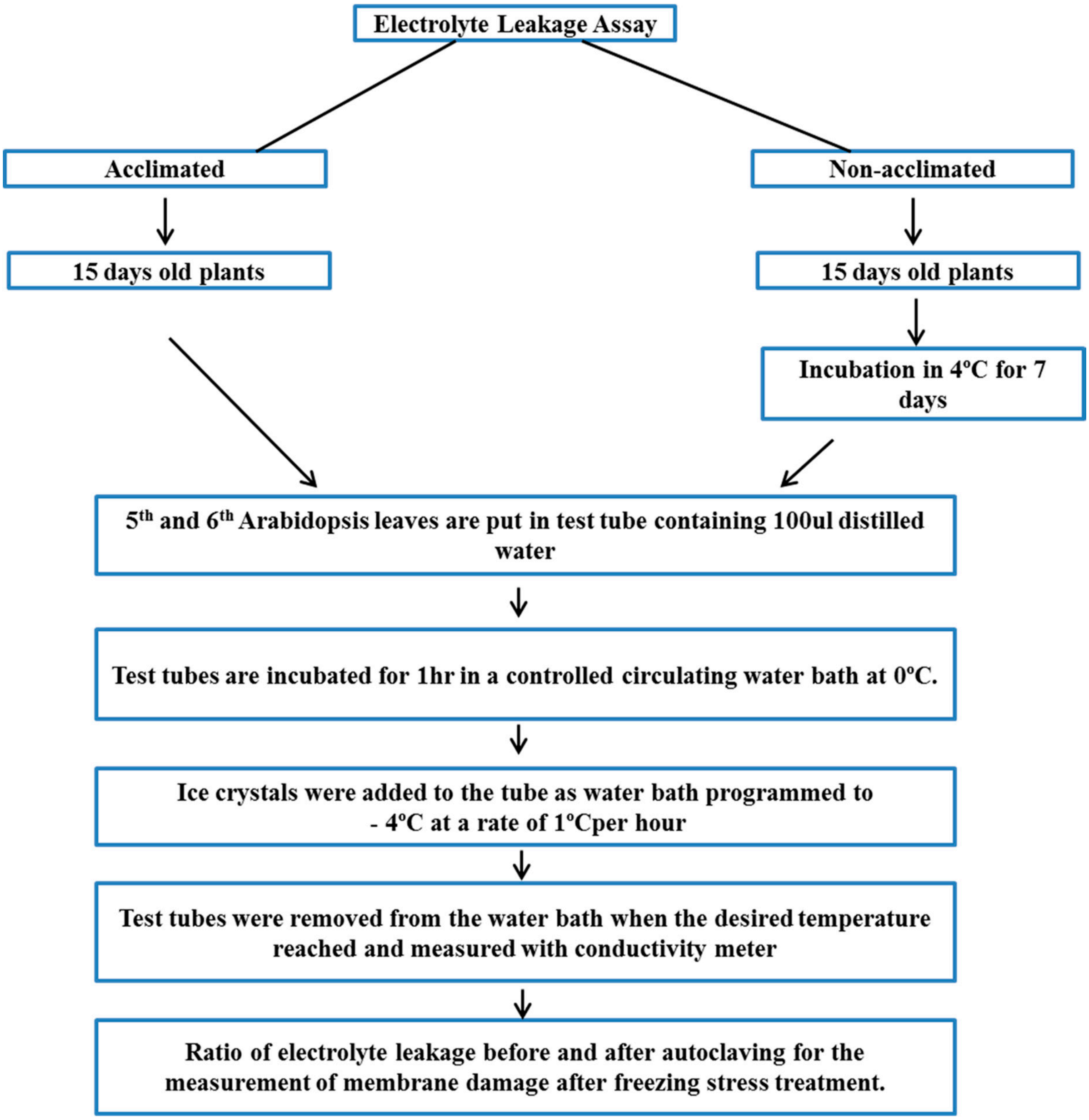

Figure S3. Schematic diagram of electrolyte leakage assay.
